# *Artemisia argyi* Extracts Modulate Prolactin and Prostaglandin Pathways in Estrogen‐Stimulated GH3 Cells and a Hyperprolactinemia Mouse Model

**DOI:** 10.1002/fsn3.71764

**Published:** 2026-04-09

**Authors:** Min‐Jung Ma, Jeonghoon Lee, JaeWan Park, Dongkyun Son, Hye‐Jin Kim, Young‐Hee Jo, Seul Bi Lee, Gyoung‐deuck Kim, En‐Jin Choi, Min‐Soo Seo, Joo‐Hee Choi

**Affiliations:** ^1^ College of Veterinary Medicine Kyungpook National University Daegu Republic of Korea; ^2^ Department of Herbal Crop Research National Institute of Horticultural & Herbal Science, RDA Republic of Korea; ^3^ Food Science R&D Center, Kolmar BNH CO., Ltd. Seoul Republic of Korea; ^4^ Preclinical Research Center Daegu‐Gyeongbuk Medical Innovation Foundation Daegu Republic of Korea

**Keywords:** *Artemisia agryi*, hyperprolactinemia, inflammation, premenstrual syndrome, prostaglandins, Pyeong‐an‐ae

## Abstract

*Artemisia argyi* (Pyeong‐an‐ae) has long been used in traditional medicine for gynecological conditions; however, its efficacy in treating premenstrual syndrome (PMS) remains underexplored. This study investigated the effects of *A. argyi* extracts on prolactin secretion, prostaglandin regulation, and inflammatory responses associated with PMS. Extracts were prepared using water and ethanol at various temperatures and screened in estradiol‐stimulated GH3 cells. Notably, a 95°C water extract and 50°C 50% ethanol extract significantly suppressed prolactin secretion and modulated PGE1 and PGE2 levels without exhibiting cytotoxicity. These two extracts were further evaluated in a mouse model of metoclopramide‐induced hyperprolactinemia. The water extract significantly reduced prolactin levels; restored estradiol, follicle‐stimulating hormone, and luteinizing hormone concentrations; and improved uterine and inflammatory markers, comparable to the effects of bromocriptine treatment. Additionally, *A. argyi* extracts altered prostaglandin balance and decreased interleukin (IL)‐1β and IL‐6 expression in uterine tissue. These findings suggest that *A. argyi*, particularly its water extract, may provide a promising natural therapeutic option for managing PMS symptoms through endocrine and inflammatory regulation.

## Graphical abstract

Artemisia argyi extracts suppress prolactin secretion, restore reproductive hormones, and modulate prostaglandin balance and inflammation in GH3 cells and a hyperprolactinemia mouse model.

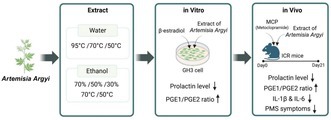

## 1 Introduction

Premenstrual syndrome (PMS), a common disorder affecting women of reproductive age, is characterized by a wide spectrum of emotional, behavioral, and physical symptoms. These symptoms typically emerge during the luteal phase of the menstrual cycle, when progesterone levels rise, and gradually subside with the onset of menstruation (Siminiuc and Ţurcanu 2023; Yonkers and Simoni 2018). Epidemiological studies estimate that PMS affects approximately 47.8% of women of reproductive age, while its severe form, premenstrual dysphoric disorder, affects approximately 3%–8% of this population (Modzelewski et al. 2024). Although PMS is generally not considered a pathological condition, its symptoms can significantly interfere with daily functioning and overall quality of life, underscoring the need for safe and effective management strategies.

The underlying pathophysiology of PMS has not been fully elucidated. However, several contributing factors have been identified, including endocrine dysregulation, aberrant prolactin secretion, altered prostaglandin activity, genetic predisposition, and medication use (Abbara et al. 2018; Hofmeister and Bodden 2016; Mann 2011). Accumulating evidence indicates that hormonal fluctuations, particularly in estradiol and progesterone levels, are strongly associated with the manifestation of PMS symptoms (Osorio et al. 2018; Yen et al. 2018). Estrogen promotes the proliferation of various pituitary cell types and enhances prolactin synthesis (Sarkar et al. 1998). Prolactin plays essential roles in gonadal function, reproduction, and lactation, and its secretion increases in response to physiological stress (Lennartsson and Jonsdottir 2011). Hyperprolactinemia has been linked to PMS‐related symptoms, such as premenstrual breast tenderness, weight gain, and mood disturbances (Majumdar and Mangal 2013; Pałubska et al. 2017). Animal models of hyperprolactinemia are frequently used to investigate PMS‐related mechanisms (Amaral et al. 2013). This model replicates several PMS‐like phenotypes, including elevated inflammatory responses, increased prostaglandin production, and thickening of the endometrial lining, rendering it a valuable tool for evaluating potential therapeutic interventions (Borba et al. 2018; Prigent‐Tessier et al. 1996). Although bromocriptine is a Food and Drug Administration‐approved and widely used dopamine agonist for the treatment of hyperprolactinemia (Krysiak et al. 2018), clinical limitations associated with its use have generated interest in identifying safer, natural alternatives.

*Artemisia agryi*, a traditional medicinal herb extensively used in East Asia, has long been used to treat gynecological disorders, including dysmenorrhea and menstrual irregularities (Adams et al. 2012). Its pharmacological properties include anti‐inflammatory, antioxidant, and hormone‐modulating effects, suggesting its potential utility in managing PMS‐related symptoms (Hu et al. 2021; Shin et al. 2021).

Recent studies have revealed that *A. argyi* polysaccharides can elevate plasma estrogen levels in ovariectomized rats, supporting their estrogenic regulatory capacity (Zhang et al. 2023). Furthermore, bioactive compounds in *A. argyi*, such as eupatilin, have been identified as selective peroxisome proliferator‐activated receptor alpha agonists, implicating additional endocrine‐related mechanisms (Choi et al. 2015). Extracts from *A. argyi* have also demonstrated antiproliferative effects on hormone‐sensitive breast cancer cells, further supporting their interaction with estrogen signaling pathways (Choi and Kim 2013). Despite these promising properties, experimental evidence regarding the effects of *A. argyi* in the context of hyperprolactinemia and PMS remains limited.

Therefore, this study aimed to evaluate the regulatory effects of *A. argyi* extracts on prolactin secretion, prostaglandin production, and inflammation using both an estrogen‐stimulated GH3 cell model and a metoclopramide (MCP)‐induced hyperprolactinemia mouse model to assess their potential as natural therapeutic options for PMS‐related symptoms.

## 2 Material and Methods

### 2.1 Preparation of Extracts

The *Artemisia argyi* used in this sutudy was a newly developed cultivar named “*Pyeong‐an‐ae*” (Registration No. 398), bred by the Department of Herbal Crop Research, National Institiute of Horticultural and Herbal Science, Rural Development Administraion, Korea. The aerial parts of *A. argyi* (Pyeong‐an‐ae) were washed and dried before extraction. The dried aerial parts of *A. argyi* were subjected to extraction twice for 4 and 2 h. The extraction solvents included water, 30% ethanol, 50% ethanol, and 70% ethanol, and the extraction temperatures were 95°C, 70°C, and 50°C (Table 1).

**TABLE 1:** List of extraction conditions.

No	Solvent	Temp
1	Water	95°C
2	Water	70°C
3	Water	50°C
4	30% Ethanol	70°C
5	50% Ethanol	70°C
6	70% Ethanol	70°C
7	30% Ethanol	50°C
8	50% Ethanol	50°C
9	70% Ethanol	50°C

### 2.2 Cell Culture

The GH3 cell line (American Type Culture Collection), derived from the pituitary gland of a female rat with a tumor, was cultured in Dulbecco's modified Eagle medium with high glucose (HyClone) supplemented with 10% (v/v) horse serum (Gibco) and 1% (v/v) penicillin–streptomycin (P/S) (Gibco) and maintained at 37°C in a 5% CO_2_‐humidified incubator.

### 2.3 Cell Viability Assay

GH3 cells (2 × 10^5^ cells/well) were seeded into a 96‐well plate and incubated overnight. Thereafter, the cells were incubated with 50 μg/mL of *A. argyi* extracts for 45 h. After treatment with the Cell Counting Kit‐8 (CCK‐8) reagent, the cells were incubated for an additional 3 h at 37°C under 5% CO_2_, and absorbance was measured at 450 nm using a microplate reader. To correct for potential color interference from the extracts, background absorbance was measured in wells containing the extract and CCK‐8 reagent without cells and subtracted from the corresponding sample readings.

### 2.4 Prolactin and Prostaglandin Measurement in GH3 Cells

GH3 cells (2 × 10^5^ cells/well) were seeded into a 24‐well plate and incubated overnight. The cells were treated with 50 μg/mL of *A. argyi* extracts in the presence of 1 nM β‐estradiol (E2, Sigma) and subsequently incubated for 48 h under standard conditions (37°C, 5% CO_2_). After incubation, the culture supernatants were collected for enzyme‐linked immunosorbent assay (ELISA) analysis. Prolactin, prostaglandin E1 (PGE1), and prostaglandin E2 (PGE2) levels were quantified using ELISA kits from Cusabio Biotech Co. Ltd. (Wuhan, China) and MyBioSource (San Diego, CA, USA), according to the manufacturer's instructions.

### 2.5 Animal Model of Hyperprolactinemia

Female ICR mice (10 weeks old, *n* = 56) were purchased from Samtako Inc. (Kyoung Gi‐Do, Korea). All animals were housed in individually ventilated cages under controlled environmental conditions (temperature: 21°C ± 2°C, humidity: 50% ± 20%, and a 12‐h light/dark cycle), with free access to food and water. After 13 days of adaptation, the mice were randomly divided into seven groups, with eight mice per group: (G1) control with vehicle, (G2) MCP with vehicle, (G3) MCP with the aqueous extract of *Artemisia argyi* (AAW) at 250 mg/kg, (G4) MCP with AAW at 500 mg/kg, (G5) MCP with the alcoholic extract of *Artemisia argyi* (AAE) at 250 mg/kg, (G6) MCP with AAE at 500 mg/kg, and (G7) MCP with bromocriptine (2 mg/kg). MCP was intraperitoneally administered at a dose of 20 mg/kg every 2 days for 21 days, while AAW, AAE, and the vehicle were orally administered once daily. AAW and AAE were prepared in 0.5% methyl cellulose (MC), whereas bromocriptine and MCP were dissolved in saline. Body weight was measured weekly throughout the experiment. At the conclusion of the experiment, the mice were fasted and euthanized, and blood and uterine samples were collected. The collected blood samples were placed in serum separator tubes (Microtainer, BD, USA) and allowed to clot at room temperature for 30 min. The samples were subsequently centrifuged at 12,000 rpm and 4°C for 5 min to separate the serum. The separated serum and uterine tissue samples were stored at −80°C for further analysis. Additionally, a portion of the uterine tissue was fixed in 10% neutral buffered formalin (NBF) for histological analysis. All animal experimental protocols were reviewed and approved by the Institutional Animal Care and Use Committee of NDIC Co. Ltd. (approval no. P241006), and all procedures complied with the relevant guidelines and regulations.

### 2.6 Histological Analysis of Uterine Tissue

Uterine tissues were fixed in 10% NBF (BBC Biochemicals, WA, USA) and processed using a tissue processor (Thermo Fisher Scientific Inc., Runcorn, UK). Paraffin‐embedded samples were sectioned to a thickness of 4 μm and mounted onto glass slides. The sections were stained with hematoxylin and eosin (H&E) using an autostainer (Dako CoverStainer; Agilent, CA, USA). Histological assessments, including the measurement of endometrial thickness, were performed under light microscopy by personnel blinded to group allocation.

Uterine tissues were carefully harvested and subsequently fixed in 10% NBF (BBC Biochemicals, WA, USA). The fixed tissues were processed using a tissue processor (Thermo Fisher Scientific Inc., Runcorn, UK). Paraffin‐embedded samples were sectioned to a thickness of 3–4 μm, mounted onto glass slides, and stained with H&E for general histopathological examination using an autostainer (Dako CoverStainer; Agilent, CA, USA). Histological evaluations, including the measurement of endometrial thickness, were conducted under light microscopy by an experienced histopathologist using computer‐assisted automated image analysis software (Nikon Eclipse software; Nikon Instruments, Tokyo, Japan).

### 2.7 Gene Expression Analysis in Uterine Tissue

Total RNA was extracted from mouse uterine tissues using TRIzol reagent (Thermo Fisher Scientific, Waltham, MA, USA). Briefly, the tissues were lysed with TRIzol, and chloroform was added to facilitate phase separation. The RNA‐containing aqueous phase was collected, and RNA was precipitated with isopropanol. The RNA pellet was subsequently washed with ethanol, air‐dried, and dissolved in RNase‐free water. RNA quality and concentration were assessed using a NanoDrop spectrophotometer (Thermo Fisher Scientific, Wilmington, DE, USA). For cDNA synthesis, 1 μg of isolated RNA was reverse transcribed using an RT premix (Toyobo Biotechnology, Osaka, Japan), following the manufacturer's instructions. Quantitative real‐time PCR (qPCR) was conducted using Takara TB Green Premix (Takara, Shiga, Japan) and gene‐specific primers (Tables 1 and 2).

**TABLE 2:** Primer (mouse) sequences for real time PCR.

Primer	Primer	Sequences
IL‐1β	Forward Reverse	ATGCCACCTTTTGACAGTGATG GCAGCCCTTCATCTTTTGGG
IL‐6	Forward Reverse	GTTCTCTGGGAAATCGTGGA TGTACTCCAGGTAGCTA
COX‐2	Forward Reverse	CTGTATCCCGCCCTGCTGGTG ACTTGCGTTGATGGTGGCTGTCTT
TNF‐α	Forward Reverse	GAAGTTCCCAAATGGCCTCC TTTGCTACGACGTGGGCTAC
β‐actin	Forward Reverse	AGGCCCAGAGCAAGAGAG TCAACATGATCTGGGTCATC

### 2.8 Measurement of Serum Hormones and Prostaglandins

Serum hormone levels were measured using ELISA kits, according to the manufacturer's protocols. Prolactin and 17β‐estradiol levels were quantified using ELISA kits from Abcam (Cambridge, UK), while progesterone, PGE1, and PGE2 concentrations were measured using kits from ENZO Life Sciences (Farmingdale, NY, USA). Follicle‐stimulating hormone (FSH) and luteinizing hormone (LH) levels were determined using ELISA kits from Wuhan Finetest Co. Ltd. (Wuhan, China). For serum analysis, samples were diluted appropriately to ensure that measured values fell within the quantitative range of each assay (prolactin, 1:20; progesterone, 1:300; PGE1, 1:15; PGE2, 1:600; estradiol, 1:1; FSH, 1:5; and LH, 1:5). Absorbance was measured using a microplate reader, and all assays were performed in accordance with the manufacturer's instructions.

### 2.9 Statistical Analysis

Data were statistically analyzed using Student's *t*‐test. Statistically significant differences between the experimental and control groups were determined using GraphPad Prism (version 5.01; GraphPad Software, San Diego, CA, USA). Statistical significance was set at *p* < 0.05.

## 3 Results

### 3.1 Effects of *A. argyi* Extracts on Prolactin and PGE Secretion in GH3 Cells

**FIGURE 1:**
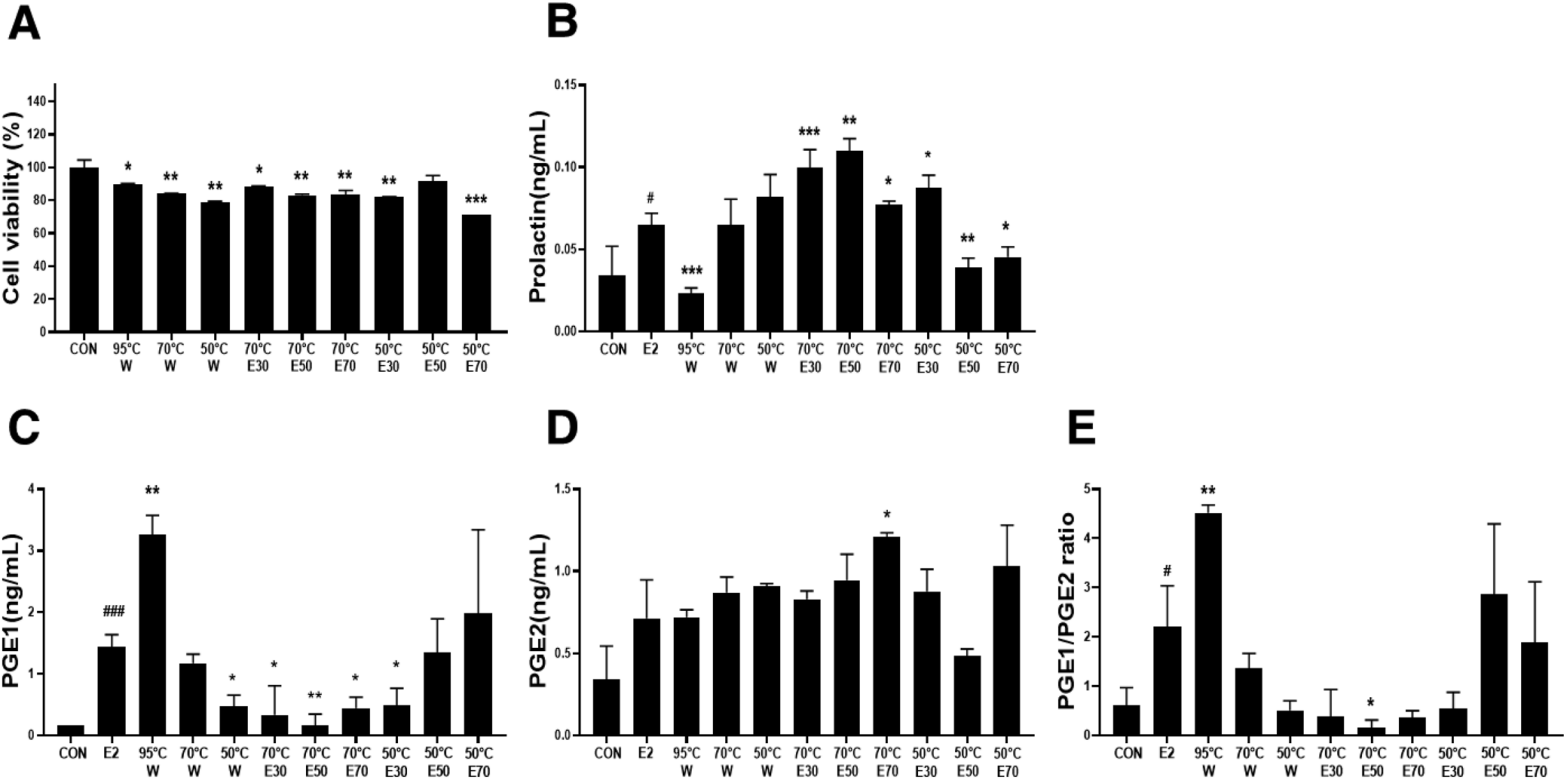
Effects of *A. argyi* extracts on cell viability and prolactin and prostaglandin levels in GH3 cells. (A) Cell viability was assessed in GH3 cells treated with *A. argy*i extracts (50 μg/mL) prepared under various extraction conditions (95°C, 70°C, and 50°C) using water (W) or ethanol (E). (B–E) GH3 cells were stimulated with 17β‐estradiol (E_2_, 1 nM) in the presence or absence of *A. argyi* extracts, and the levels of prolactin (B), PGE1 (C), PGE2 (D), and the PGE1/PGE2 ratio (E) were quantified. Data are presented as the mean ± SD. #*p* < 0.05, ###*p* < 0.001 versus CON; *p < 0.05, **p < 0.01, ***p < 0.001 versus the CON or E‐treated group.

To investigate the biological activity of *A. argyi* (Pyeong‐an‐ae) extracts prepared under various extraction conditions, GH3 cells were treated with water extracts obtained at 95°C, 70°C, and 50°C as well as ethanol extracts (30%, 50%, and 70%) prepared at 70°C and 50°C. Cytotoxicity was evaluated using the CCK‐8 assay (Figure 1A). While several treatment groups exhibited statistically significant differences in viability compared with the control, all values remained above 80%, indicating that the extracts did not exert substantial cytotoxic effects at the tested concentration (50 μg/mL).

Treatment with β‐estradiol (E2) significantly increased prolactin secretion compared with the control, confirming the responsiveness of GH3 cells. Among the extract‐treated groups, the 95°C water (0.023 ± 0.003, *p* < 0.001) and 50°C 50% ethanol (0.039 ± 0.006, *p* < 0.01) extracts significantly suppressed E2‐induced prolactin levels (0.065 ± 0.007, *p* < 0.05), suggesting inhibitory effects under specific extraction conditions (Figure 1B).

To further assess extract‐related effects on prostaglandins, PGE1 and PGE2 levels were measured (Figure 1C,D). E2 (1.436 ± 0.2, *p* < 0.001) significantly elevated PGE1 levels. The 95°C water extract further increased PGE1 levels (3.261 ± 0.31, *p* < 0.01), while the 50°C 50% ethanol (1.350 ± 0.542, n.s) and 50°C 70% ethanol (1.978 ± 1.361, n.s) extracts showed numerically higher values than control but were not significantly different from the E2 group. All other extracts significantly reduced PGE1 levels compared to E2 treatment. In contrast, PGE2 levels remained largely unchanged, except for a significant increase observed with the 70°C 70% ethanol (1.207 ± 0.027, *p* < 0.05) extract. Finally, the PGE1/PGE2 ratio, which increased with E2 treatment (2.198 ± 0.838, *p* < 0.05), was further elevated exclusively by the 95°C water extract (4.519 ± 0.157, *p* < 0.01), while all other extracts either suppressed the ratio or had no effect (Figure 1E).

### 3.2 Effects of AA Extracts on Body Weight and Uterine Histology in a Hyperprolactinemia Model

**FIGURE 2:**
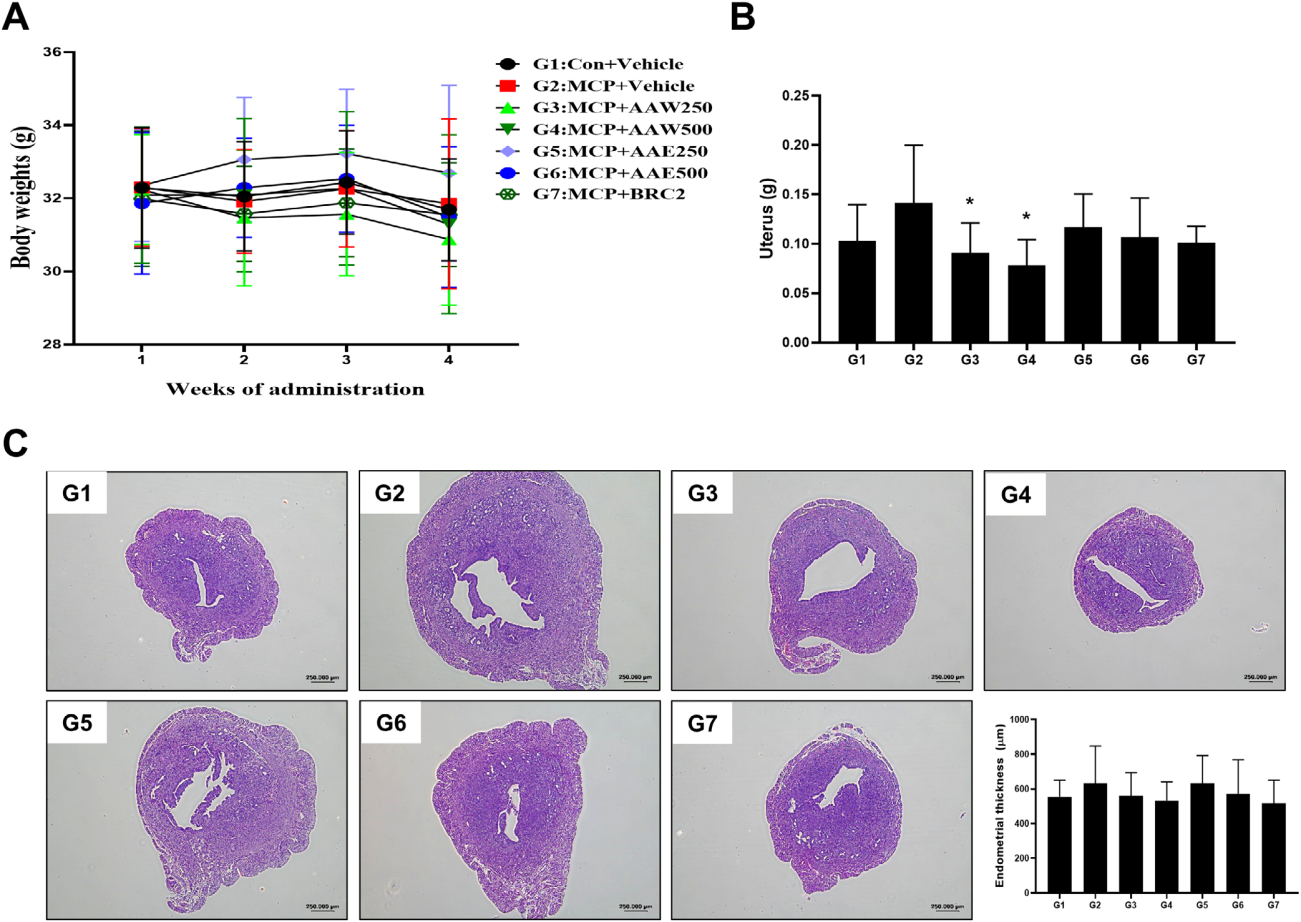
Effects of *A. argyi* extracts on body weight and uterine histology in MCP‐induced hyperprolactinemia mice. (A) Body weight changes during the 21‐day experimental period. (B) Uterine weights at the end of treatment. (C) Representative hematoxylin and eosin (H&E)‐stained uterine sections (scale bar: 250 μm) and quantitative analysis of endometrial thickness. Hyperprolactinemia was induced via metoclopramide (MCP, 20 mg/kg) injection, and mice were treated with *A. argyi* water (AAW) or ethanol (AAE) extracts (250 and 500 mg/kg each), or bromocriptine (BRC, 2 mg/kg) as a positive control. Data are expressed as the mean ± SD (*n* = 8). *p < 0.05 versus G2 (MCP + vehicle).

Based on the in vitro results indicating that the 95°C water and 50°C 50% ethanol extracts of *A. argyi* effectively modulated prolactin and PGE1 levels, these extracts were finally subjected to further evaluation in an in vivo model of hyperprolactinemia. Hyperprolactinemia was induced in mice via intraperitoneal injection of metoclopramide (20 mg/kg) every other day. The 95°C water (AAW) and 50°C 50% ethanol (AAE) extracts were orally administered at doses of 250 and 500 mg/kg each. Bromocriptine served as a positive control (Krysiak et al. 2018). Body weight was monitored throughout the experimental period, with no significant differences observed among the experimental groups (Figure 2A). At the conclusion of the experiment, uterine tissues were collected for weight measurement and histological analysis. Uterine weight increased in the MCP‐induced group (0.141 ± 0.058, n.s) compared with that in the control group (0.103 ± 0.036), although the difference was not statistically significant. Compared with the MCP‐induced group, AAW extract significantly reduced uterine weight at both 250 mg/kg (0.091 ± 0.030, *p* < 0.05) and 500 mg/kg (0.078 ± 0.026, *p* < 0.05) (Figure 2B). In contrast, no statistically significant changes in uterine weight were observed in the AAE extract‐treated or the bromocriptine‐treated group compared with the MCP‐induced group. Thereafter, endometrial thickness was measured to assess histological changes (Figure 2C). The MCP‐induced group (632.04 ± 215.47) showed a higher mean endometrial thickness than the control group (555.44 ± 215.47); however, this difference was not statistically significant. Compared with the MCP‐induced group, no statistically significant differences in endometrial thickness were observed in the AAW extract‐treated groups (559.57 ± 134.70 and 531.84 ± 108.54), the AAE extract‐treated groups (570.94 ± 197.50 and 517.66 ± 133.23), or the bromocriptine‐treated group (517.66 ± 133.23).

### 3.3 Effects of AA Extracts on Serum Hormone Levels in a Hyperprolactinemia Model

**FIGURE 3:**
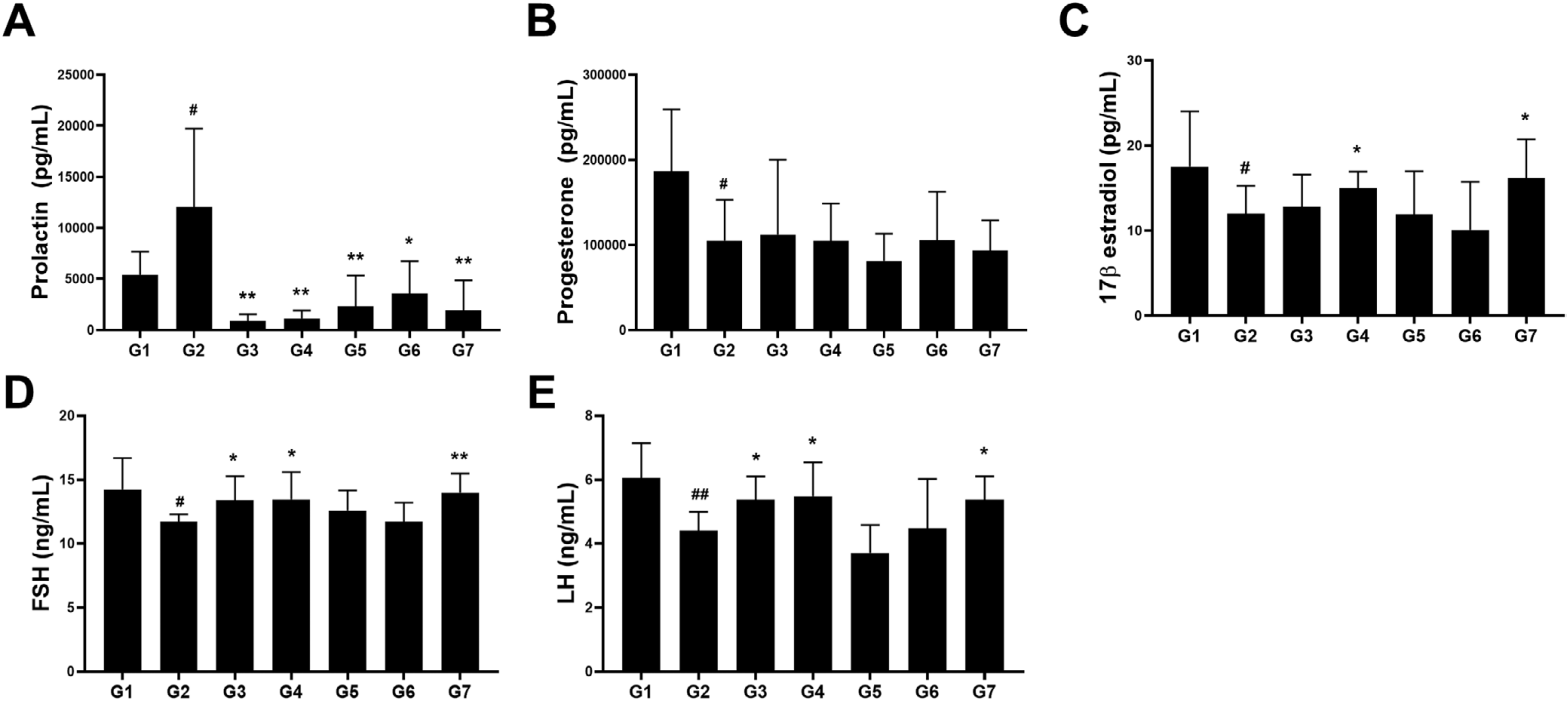
Effects of *A. argyi* extracts on serum prolactin and reproductive hormone levels in MCP‐induced hyperprolactinemia mice. Serum concentrations of (A) prolactin, (B) progesterone, (C) 17β‐estradiol, (D) follicle‐stimulating hormone (FSH), and (E) luteinizing hormone (LH) were measured at the conclusion of the experiment. Data are expressed as the mean ± SD (*n* = 8). ^#^ *p* < 0.05, ##*p* < 0.01 versus G1 (control); *p < 0.05, **p>*p* < 0.01 versus G2 (MCP + vehicle).

Serum prolactin, progesterone, 17β‐estradiol, FSH, and LH levels were measured using ELISA kits (Figure 3). Serum prolactin levels were measured to confirm the induction of hyperprolactinemia by MCP. The MCP‐induced group (12,018.8 ± 7709.8, *p* < 0.05) exhibited a significant increase in prolactin levels compared with the control group (5404.9 ± 2257.6). Treatment with AA extracts and bromocriptine significantly reduced prolactin levels (Figure 3A). Notably, AAW treatment at both 250 mg/kg (853.1 ± 679.0, *p* < 0.01) and 500 mg/kg (1114.3 ± 800.2, *p* < 0.01) significantly suppressed serum prolactin level, indicating a strong inhibitory effect. Serum progesterone levels decreased in the MCP‐induced group (105,037 ± 47,863.8, *p* < 0.05) compared with those in the control group (186,706.4 ± 72,397.2); nevertheless, no significant changes were observed in the other treatment groups (Figure 3B). Conversely, MCP induction reduced serum 17β‐estradiol, FSH, and LH levels (Figure 3C–E). Serum 17β‐estradiol levels were lower in the MCP‐induced group (11.97 ± 3.27, *p* < 0.05) but increased following AAW treatment at 500 mg/kg (14.97 ± 1.96, *p* < 0.05), and were further elevated by bromocriptine treatment (16.20 ± 4.54, *p* < 0.05). FSH levels were significantly reduced in the MCP‐induced group (11.72 ± 0.58, *p* < 0.05) compared with the normal control group (14.33 ± 2.28) and were restored toward normal levels by AAW treatment at 250 and 500 mg/kg (13.39 ± 1.89, *p* < 0.05) and 500 mg/kg (13.48 ± 2.14, *p* < 0.05), as well as by bromocriptine (14.00 ± 1.50, *p* < 0.01). Similarly, LH levels were decreased in the MCP group (4.41 ± 0.59, *p* < 0.01) relative to the normal control (6.03 ± 1.12) and were restored toward control values by AAW 250 and 500 mg/kg (5.38 ± 0.72, *p* < 0.05, and 5.49 ± 1.05, *p* < 0.05) and bromocriptine (5.38 ± 0.73, *p* < 0.05). These findings suggest that AAW treatment alleviated the MCP‐induced suppression of serum reproductive hormones.

### 3.4 Effects of AA Extracts on Prostaglandin Levels in a Hyperprolactinemia Model

**FIGURE 4:**
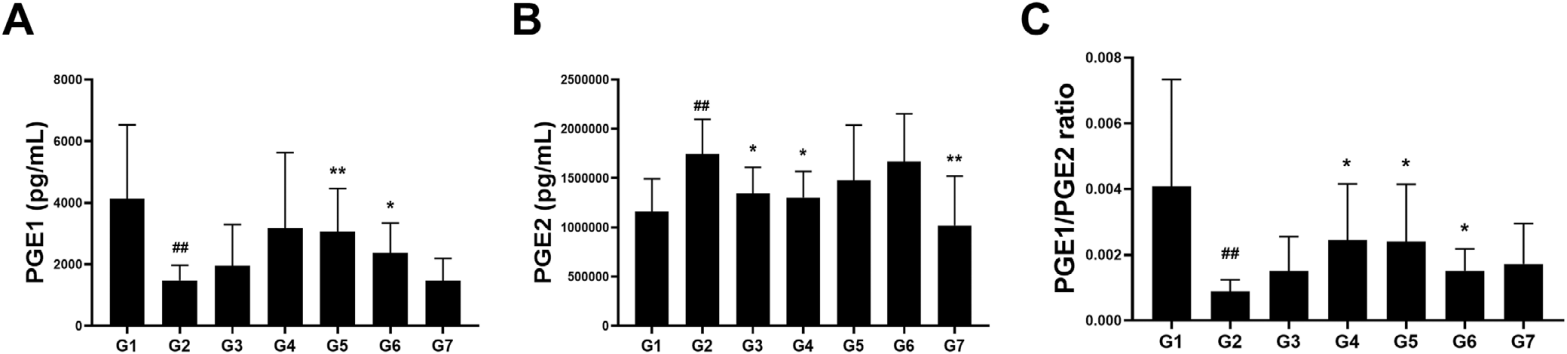
Effects of *A. argyi* extracts on serum prostaglandin levels in MCP‐induced hyperprolactinemia mice. Serum levels of (A) PGE_1_, (B) PGE_2_, and (C) the PGE_1_/PGE_2_ ratio were analyzed after treatment. Data are presented as the mean ± SD (*n* = 8). ##*p* < 0.01 versus G1 (control); *p < 0.05, **p < 0.01 versus G2 (MCP + vehicle).

Serum PGE1 and PGE2 levels were measured to assess the impact of AA extracts on prostaglandin regulation in hyperprolactinemia (Figure 4). PGE1 levels significantly decreased in the MCP‐induced group (1480.7 ± 494, *p* < 0.01) compared with those in the control group (4120.5 ± 2407.5). AAE (3072.9 ± 1391.8, *p* < 0.01; 2374.8 ± 967.5, *p* < 0.05) significantly increased PGE1 levels, albeit not dose‐dependently. AAW (1960.6 ± 1333.1, n.s; 3168.6 ± 2458.2, n.s) exhibited a dose‐dependent, yet not statistically significant, increasing trend. Bromocriptine (1473.2 ± 713.9, n.s) treatment demonstrated no significant difference compared with MCP treatment (Figure 4A). Conversely, the MCP‐induced group (1,742,445.5 ± 354,909, *p* < 0.01) exhibited elevated PGE2 levels. AAW (1,342,343.1 ± 266,341.5, *p* < 0.05; 1302,235.7 ± 265,432.1, *p* < 0.05) and bromocriptine (1,021,154.7 ± 497,954.4, *p* < 0.01) significantly suppressed this increase, while AAE exerted no significant effect (Figure 4B). The PGE1/PGE2 ratio was significantly restored in the high‐dose AAW and low‐dose AAE groups (Figure 4C).

### 3.5 Effects of AA Extracts on Gene Expression in Uterine Tissue

**FIGURE 5:**
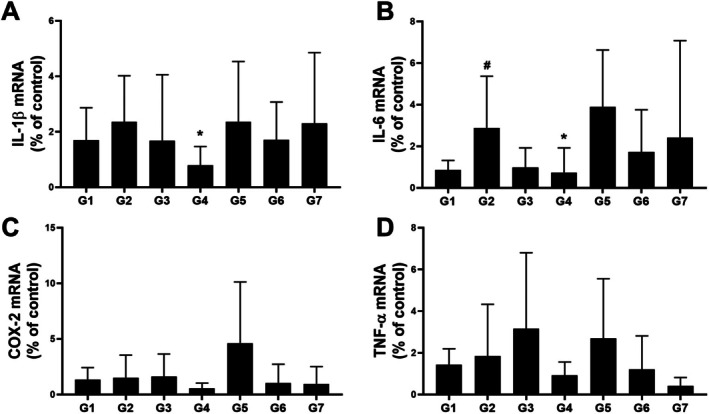
Effects of *A. argyi* extracts on the uterine mRNA expression of inflammatory genes in MCP‐induced hyperprolactinemia mice. The mRNA expression levels of (A) IL‐1β, (B) IL‐6, (C) COX‐2, and (D) TNF‐α in uterine tissue were quantified using real‐time PCR. Gene expression levels were normalized to β‐actin. Data are presented as the mean ± SD (*n* = 8). #*p* < 0.05 versus G1 (control); *p < 0.05 versus G2 (MCP + vehicle).

Considering that hyperprolactinemia has been associated with inflammatory responses, we examined the expression of inflammatory genes in uterine tissue. Interleukin (IL)‐1β mRNA expression exhibited a non‐significant increasing trend in the MCP‐induced group (2.36 ± 1.66, n.s) compared with that in the control group (1.7 ± 1.17), while IL‐6 expression (2.89 ± 2.48, *p* < 0.05) significantly increased. Notably, IL‐1β and IL‐6 expression levels significantly decreased in the high‐dose AAW group (0.79 ± 0.68, *p* < 0.05; 0.74 ± 1.18, *p* < 0.05) compared with those in the MCP group (Figure 5A,B). No significant differences in cyclooxygenase‐2 (COX‐2) and tumor necrosis factor‐alpha (TNF‐α) mRNA expression were observed among the groups (Figure 5C,D).

## 4 Discussion

PMS is a widespread condition characterized by both physical and psychological symptoms that occur during the luteal phase of the menstrual cycle. Its pathophysiology is multifactorial, with prolactin dysregulation and inflammatory responses recognized as key contributors. Although pharmacological treatments are available, their side effects often limit long‐term use (Kim et al. 2024; Saglam and Orsal 2020; Freeman 2002). Considering the traditional application of *A. argyi* (Pyeong‐an‐ae) in gynecological disorders and its reported endocrine and anti‐inflammatory properties (Adams et al. 2012; Zhang et al. 2023), we hypothesized that *A. argyi* extracts may alleviate PMS‐related symptoms. Our findings support this hypothesis, demonstrating that selected extracts of *A. argyi* modulate prolactin secretion, prostaglandin balance, and inflammatory gene expression in both estrogen‐stimulated GH3 cells and an MCP‐induced hyperprolactinemia mouse model.

To identify optimal extraction conditions for the bioactive constituents of *A. argyi*, we prepared extracts using various water‐ and ethanol‐based solvents at different temperatures. The ability of these extracts to modulate prolactin secretion induced by estradiol stimulation—which reflects the endocrine imbalance prevalently observed in PMS—was subsequently evaluated in GH3 cells (Ye et al. 2010).

Estradiol enhances prolactin secretion in GH3 cells through the estrogen receptor‐mediated activation of prolactin gene transcription (Ying and Lin 2000) and increased intracellular calcium influx via voltage‐gated calcium channels (Davis 1990; Sánchez et al. 2017). Consistently, our in vitro experiments confirmed that 17β‐estradiol significantly increased prolactin levels in GH3 cells, validating the estrogen‐mediated prolactin regulation model. Among the various *A. argyi* extracts, the 95°C water and 50°C 50% ethanol extracts significantly suppressed E2‐induced prolactin secretion, indicating their potential inhibitory effects on estrogen‐responsive prolactin pathways. While GH3 cells are not typically employed to study prostaglandin secretion, measurable changes in PGE1 and PGE2 were observed following estrogen and extract treatment. Notably, the 95°C water extract of *A. argyi* further elevated PGE1 levels beyond those induced by estrogen alone. Considering the established role of PGE1 in attenuating prolactin activity and modulating inflammatory responses, this finding suggests an additional mechanism by which the extract alleviates PMS symptoms (Horrobin 1983). In contrast, the 50°C 50% ethanol extract exhibited a non‐significant trend toward reduced PGE2 secretion, which is noteworthy considering the role of PGE2 in promoting pain, inflammation, and uterine contractions associated with PMS (Kim et al. 2024). Although the mechanistic basis warrants further investigation, these patterns suggest the differential regulation of prostaglandins by *A. argyi* contingent upon extraction conditions. Following in vitro screening, the 95°C water and 50°C 50% ethanol extraction conditions were selected for in vivo evaluation using a hyperprolactinemia mouse model.

Hyperprolactinemia is considered a contributing factor to PMS, as elevated prolactin levels potentially disrupt reproductive hormone balance and trigger typical PMS symptoms (Halbreich 2003). To model this condition in vivo, hyperprolactinemia was induced in mice by administering MCP, a dopamine D_2_ receptor antagonist that promotes prolactin secretion (Rossi et al. 2003). To investigate the effects of *A. argyi* in vivo, hyperprolactinemia was induced via intraperitoneal injection of metoclopramide (20 mg/kg) every other day for 21 days. Bromocriptine, a dopamine agonist clinically used to treat PMS‐related hyperprolactinemia, was utilized as a positive control (Ye et al. 2010). Although MCP‐induced hyperprolactinemia did not alter body weight, it increased uterine weight and endometrial thickening, as evidenced by histological analysis. These alterations were attenuated by treatment with the AAW extract. This observation aligns with previous reports indicating that MCP‐induced hyperprolactinemia is associated with endometrial thickening, a condition that may exacerbate PMS‐related symptoms, such as pelvic discomfort and menstrual irregularities (Kim et al. 2024; Rossi et al. 2003). The post‐treatment restoration of normal endometrial architecture suggests that the AAW extract may alleviate PMS‐related uterine abnormalities by mitigating prolactin‐induced histological alterations (Kang et al. 2022). Considering the significance of the endometrial changes associated with PMS symptoms, particularly dysmenorrhea and abnormal bleeding, the ability of *A. argyi* to normalize uterine morphology further corroborates its therapeutic relevance in PMS management. Additionally, MCP‐induced elevations in prolactin levels are known to disrupt the hypothalamic–pituitary–gonadal axis, reducing the secretion of gonadotropins, such as FSH and LH, as well as ovarian hormones, including estrogen and progesterone (Abbara et al. 2018). Consistent with the hormonal disruptions typically observed in hyperprolactinemia, our study confirmed that MCP administration reduced serum progesterone, estradiol, FSH, and LH levels. Notably, treatment with *A. argyi* extracts, particularly the water extract, significantly lowered prolactin levels and restored estradiol, FSH, and LH levels to values comparable to those achieved with bromocriptine. The distinct effects of the aqueous and ethanol extracts on uterine mass and reproductive hormones are likely attributable to solvent‐dependent differences in phytochemical composition. Water extraction preferentially enriches polar bioactive compounds, including polysaccharides and glycosylated flavonoids, whereas ethanol extracts contain relatively higher proportions of lipophilic phenolics and aglycones (Do et al. 2014). Notably, polysaccharides derived from *A. argyi* have been shown to increase circulating estrogen levels and regulate reproductive homeostasis in ovariectomized rats (Zhang et al. 2023), supporting the concept that water‐soluble constituents contribute to endocrine normalization. In addition, plant‐derived polysaccharides and hydrophilic phytochemicals exert anti‐inflammatory and immunomodulatory activities, including suppression of IL‐1β and IL‐6, which are known to interfere with prolactin‐dependent disruption of the hypothalamic–pituitary–gonadal axis (Abbara et al. 2018; Schepetkin and Quinn 2006). Therefore, the superior efficacy of the AAW extract in reducing uterine hypertrophy and restoring LH and estradiol levels in this model likely reflects its enrichment in water‐soluble endocrine‐ and inflammation‐modulating constituents. Further phytochemical profiling will be required to identify the specific constituents responsible for these activities.

Elevated prolactin levels reportedly influence the production of prostaglandins, particularly PGE1 and PGE2. PGE1 functions as a regulatory mediator that alleviates PMS symptoms, and therapeutic approaches using its essential fatty acid precursors have been explored for PMS management (Horrobin 1983). In contrast, PGE2 is known to promote PMS‐related inflammation, pain, and uterine contractions (Koshikawa et al. 1992). Prolactin has also been shown to increase the expression of secretory phospholipase A2 group IIA and prostaglandin–endoperoxide synthase 2 in uterine cells, culminating in enhanced PGE2 biosynthesis (Prigent‐Tessier et al. 1996). Previous studies using MCP‐induced hyperprolactinemia mouse models have demonstrated that natural compounds effectively reduce elevated PGE2 levels, contributing to the amelioration of PMS‐related inflammatory and nociceptive symptoms (Lee et al. 2021). In line with these reports, MCP‐induced hyperprolactinemia in the present study was associated with a prostaglandin imbalance characterized by decreased PGE1 levels, elevated PGE2 levels, and a reduced PGE1/PGE2 ratio. Treatment with *Artemisia argyi* extracts modulated this imbalance in a solvent‐dependent manner. The alcoholic extract significantly increased PGE1 levels compared with the MCP‐induced group but did not effectively suppress elevated PGE2 levels. In contrast, the aqueous extract, particularly at the higher dose, significantly reduced PGE2 levels and restored them toward control values, comparable to the effect of bromocriptine. Although both aqueous and alcoholic extracts increased the PGE1/PGE2 ratio, the underlying mechanisms differed. Ratio elevation driven primarily by increased PGE1 may reflect partial restoration of regulatory prostaglandin signaling, whereas ratio normalization achieved through suppression of excessive PGE2 more directly indicates attenuation of a pro‐inflammatory uterine prostaglandin milieu. Given the established role of PGE2 in uterine inflammation and pain, suppression of excessive PGE2 production by the aqueous extract may contribute to the alleviation of PMS‐associated uterine inflammation and discomfort through normalization of the prostaglandin milieu. To further investigate the inflammatory pathways potentially involved in PMS pathophysiology, we evaluated the uterine mRNA expression of key proinflammatory mediators, including IL‐1β, IL‐6, COX‐2, and TNF‐α. These genes are intricately linked to prostaglandin signaling and are reportedly modulated by prolactin and prostaglandin levels. IL‐1β and IL‐6 are key proinflammatory cytokines that enhance prostaglandin synthesis by upregulating phospholipase A2 and COX‐2, amplifying inflammatory signaling (Kozawa et al. 1998; Xue et al. 1995). COX‐2, an inducible enzyme, catalyzes the conversion of arachidonic acid to PGE2 and is often elevated in hormonally or inflammation‐activated uterine tissue (Simon 1999). TNF‐α also promotes COX‐2 expression and helps increase prostaglandin production (Arslan and Zingg 1996). In our study, MCP administration elicited a significant increase in IL‐1β and IL‐6 expression, validating their roles as downstream mediators of prolactin and prostaglandin signaling. In contrast, COX‐2 and TNF‐α levels remained unchanged, suggesting the selective activation of inflammatory pathways in this model (Williams et al. 2024). Notably, treatment with high‐dose AAW significantly reduced IL‐1β and IL‐6 expression, indicating an anti‐inflammatory effect within the uterine environment. These findings suggest that *A. argyi* may attenuate prolactin‐induced uterine inflammation by downregulating specific cytokines involved in prostaglandin‐mediated signaling.

## 5 Conclusions

In the present study, *A. argyi* (Pyeong‐an‐ae) extracts prepared under various conditions were screened in vitro, revealing that the 95°C water and 50°C 50% ethanol extracts effectively suppressed estrogen‐induced prolactin secretion in GH3 cells without exhibiting cytotoxicity. These extracts were further evaluated in vivo, where the AAW extract displayed the most favorable effects, including the suppression of prolactin, restoration of reproductive hormones, and modulation of uterine and inflammatory markers. Considering that the AAW extract consistently yielded beneficial outcomes across multiple parameters, further studies are warranted to identify and characterize its active constituents. Detailed phytochemical analyses may elucidate the specific compounds responsible for the observed endocrine and anti‐inflammatory effects.

## Author Contributions

Conceptualization: M.‐S.S. and J.‐H.C. Investigation and resources: J.L., J.P., and D.S. Methodology: M.‐J.M., H.‐J.K., S.B.L., and Y.‐H.J. Data curation and formal analysis: M.‐J.M., G.‐D.K., and E.‐J.C. Supervision and writing – original draft and review and editing: M.‐S.S. and J.‐H.C.

## Funding

This work was supported by the Cooperative Research Program for Agriculture Science and Technology Development (Project No. RS‐2022‐RD010228) Rural Development Administration, Republic of Korea.

## Ethics Statement

All animal experiments were conducted in accordance with the relevant guidelines and regulations for the care and use of laboratory animals. The study protocol was reviewed and approved by the Institutional Animal Care and Use Committee (IACUC) of NDIC Co. Ltd. (Approval No. P241006).

## Conflicts of Interest

The authors declare no conflicts of interest.

## Data Availability

The data that support the findings of this study are available from the corresponding author upon reasonable request.
